# Increase in CO_2_ concentration could alter the response of *Hedera helix* to climate change

**DOI:** 10.1002/ece3.4388

**Published:** 2018-07-30

**Authors:** Rubén D. Manzanedo, Juan Ballesteros‐Cánovas, Floris Schenk, Markus Stoffel, Markus Fischer, Eric Allan

**Affiliations:** ^1^ Institute of Plant Sciences University of Bern Bern Switzerland; ^2^ dendrolab.ch Institute of Geological Sciences University of Bern Bern Switzerland; ^3^ Climatic Change and Climate Impacts Institute for Environmental Sciences University of Geneva Geneva Switzerland; ^4^ Department of Earth Sciences University of Geneva Geneva Switzerland

**Keywords:** carbon fertilization, climate change, dendrochronology, *Hedera helix* (English ivy), spatial distribution models, tree rings

## Abstract

Increasing CO_2_ concentration ([CO_2_]) is likely to affect future species distributions, in interaction with other climate change drivers. However, current modeling approaches still seldom consider interactions between climatic factors and the importance of these interactions therefore remains mostly unexplored. Here, we combined dendrochronological and modeling approaches to study the interactive effects of increasing [CO_2_] and temperature on the distribution of one of the main European liana species, *Hedera helix*. We combined a classical continent‐wide species distribution modeling approach with a case study using *H. helix* and *Quercus cerris* tree rings, where we explored the long‐term influence of a variety of climate drivers, including increasing [CO_2_], and their interactions, on secondary growth. Finally, we explored how our findings could influence the model predictions. Climate‐only model predictions showed a small decrease in habitat suitability for *H. helix* in Europe; however, this was accompanied by a strong shift in the distribution toward the north and east. Our growth ring data suggested that *H. helix* can benefit from high [CO_2_] under warm conditions, more than its tree hosts, which showed a weaker response to [CO_2_] coupled with higher cavitation risk under high temperature. Increasing [CO_2_] might therefore offset the negative effects of high temperatures on *H. helix,* and we illustrate how this might translate into maintenance of *H. helix* in warmer areas. Our results highlight the need to consider carbon fertilization and interactions between climate variables in ecological modeling. Combining dendrochronological analyses with spatial distribution modeling may provide opportunities to refine predictions of how climate change will affect species distributions.

## 1 INTRODUCTION

Ecosystems are changing rapidly in response to increasing temperature, altered precipitation patterns, and increasing CO_2_ concentrations ([CO_2_]), all of which are influencing the abundance and distribution of many plant and animal species across the globe (Lindner et al., 2014). Positive carbon fertilization effects have been shown in experiments (Bader et al., 2013), but it is unclear whether increased carbon availability could affect the growth of natural populations. Additionally, climate change drivers may show complex long‐term interactions that can modify their effect on plant performance and species interactions (e.g. Lindner et al., 2014). However, the main tool to model species response to climate change at large scales, spatial distribution models (SDMs), seldom account for increased [CO_2_], or variable interactions, likely because this is methodologically challenging (Norby & Luo, 2004). As a consequence, it is unclear whether carbon fertilization might interact with other climate change drivers to influence the outcome of climate models.

Lianas are common in forests across the world and influence forest ecosystem functioning (Schnitzer & Bongers, 2002; Tymen et al., 2016) and carbon storage capacity (van der Heijden, Powers, & Schnitzer, 2015). They affect key ecological processes such as tree mortality, susceptibility to wind damage, compositional turnover, and species diversity (Allen, Sharitz, & Goebel, 2007; Körner, 2004; Ladwig & Meiners, 2010; Schnitzer & Bongers, 2002, 2011). Lianas may also be one group that could strongly benefit from climate change and they significantly increased in abundance and productivity in the last decades (Phillips et al., 2002; Schnitzer, 2015; Schnitzer & Bongers, 2002). The drivers behind this trend remain contentious. Liana increase was first reported in the neotropics (Phillips et al., 2002) but since then, contrasting results have been shown in temperate (Heinrichs & Schmidt, 2015) and African forests (reviewed in Schnitzer, 2015), where there have been no long‐term changes in liana abundance. European lianas, on the other hand, have attracted limited attention, other than as invasive species in North American forests (Heinrichs & Schmidt, 2015). This is likely due to the low abundance and economic impact of European liana species in forestry, to the point that they have even been dismissed as “mainly decorative” (Silvertown, 2008). However, lianas are still an important part of European forest biodiversity and their loss from forests could have cascading effects on the biodiversity of other groups. Therefore, understanding the response of European lianas to climate change is important to predict future trends in forest biodiversity.

**Figure 1:**
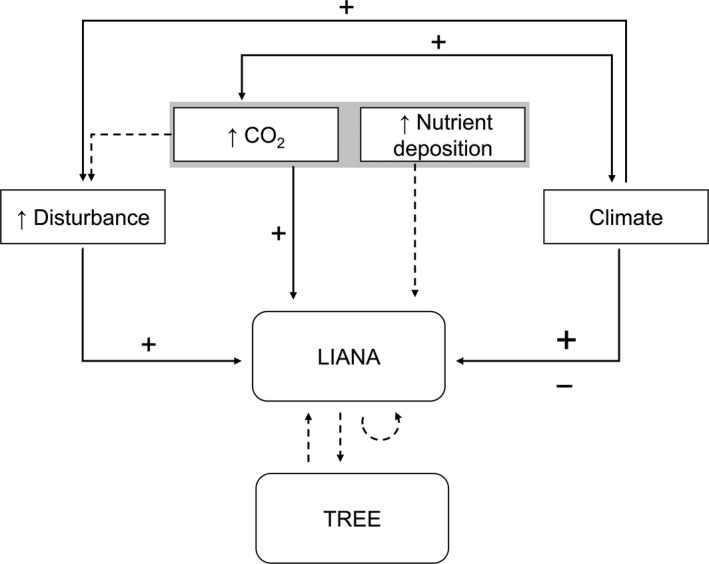
Main hypotheses for liana expansion under changing climate. “+”: suggested positive effects, “−”: suggested negative effects. Dashed lines are relationships untested in the literature. The present work focuses on the relationships: Climate–LIANA, ↑CO_2_–LIANA, and the interactive effect Climate–↑CO_2_

The most important and widespread of the liana woody species in Europe is English ivy, *Hedera helix*. There have been a handful of works studying *H. helix*'s growth, effects on host performance, and host preference (Nola 1997, Schnitzer & Bongers, 2002; Garfi & Ficarrotta, 2003; Heuzé, Dupouey, & Schnitzler, 2008; Castagneri, Garbarino, & Nola, 2013); however, it yet remains unknown how *H. helix* might respond to the new environmental conditions brought by recent and future changes in climate. Three main mechanisms have been suggested for the observed expansion of liana species in recent years: higher resource availability (which includes both increased atmospheric carbon concentration and increased nutrient deposition), changes in temperature and precipitation, and increased levels of disturbance (see Figure 1; further discussed in Schnitzer, 2005, 2015). Most of these effects, however, are still poorly explored, and only the positive correlation of liana density with disturbance is well documented in both tropical (e.g. Ledo & Schnitzer, 2014) and temperate environments (e.g. Allen et al., 2007). Some studies have explored the effects of carbon fertilization on liana growth (Hättenschwiler & Körner, 2003; Marvin, Winter, Burnham, & Schnitzer, 2015; Schnitzer, 2015; Zotz, Cueni, & Körner, 2006), but these results remain highly controversial as carbon fertilization experiments have shown results ranging from no response (Marvin et al., 2015) to over 60%–100% biomass stimulation (Hättenschwiler & Körner, 2003; Zotz et al., 2006). The effect of changing temperature is also uncertain, although it has been presumed that an increase in temperatures will likely facilitate lianas to survive winter conditions (Schnitzer, 2005). There are no comparative assessments of the importance of each mechanism and their interactions, which would be necessary to accurately forecast how these species will respond to future climatic change.

The response of *H. helix* to changing environment will likely depend on the action of several climate change drivers, which can also be affected by the interactions between them. In the case of *H. helix,* we expect both temperature and precipitation to be critical factors to define the species distribution. We also expect *H. helix* to profit from increased carbon availability. However, given the high drought stress that Mediterranean plants experience in summer, we also expect summer water availability to influence whether the plant can profit from the increased carbon availability. Specifically, we aim here to explore the following questions: (a) How might *H. helix* respond to future climate change? To address this point, we used presence records to model the species climatic niche using SDMs, to obtain predictions of suitable habitat under current and future climatic conditions. (b) Does *H. helix* benefit from increasing atmospheric CO_2_ concentrations ([CO_2_])? To explore this, we used data from a study site in Italy to look at the long‐term interactive effects of [CO_2_] and temperature on the performance of *H. helix*, and its host tree, *Quercus cerris* using tree rings and assessing cavitation risk. (c) Could carbon fertilization effects substantially modify the climate‐only model habitat predictions? As higher carbon availability cannot currently be accounted for in the SDM, we explored the potential effects by creating two future carbon fertilization scenarios for *H. helix* and comparing them with the previously developed, climate‐only model.

## 2 METHODS

### 2.1 Species distribution modeling

We constructed SDMs from publicly available occurrence data for one of the main liana species in European forests, *H. helix*. We used occurrence data from the global biodiversity information facility (GBIF, http://www.gbif.org), where we downloaded 18,265 presence records of the species. In our search, we used the species name and limited the results to “observation” data after 1900, to avoid including herbarium specimens, specimens preserved in botanical gardens, or machine observations. Repeated presence points (i.e. those with exactly same coordinates) were eliminated to avoid overfitting problems. Climate information was obtained from the Bioclim dataset (http://www.worldclim.org/bioclim), which includes 19 biologically meaningful variables derived from monthly temperature and rainfall, and is commonly used in SDM studies (Hijmans, Cameron, Parra, Jones, & Jarvis, 2005). The variables included in the Bioclim dataset are described in the Supplementary Material. Forecasted climatic values for 2050 and 2070 were obtained from downscaled predictions by the atmospheric model Hadley Global Environment Model (HadGEM2‐AO) (Martin et al., 2011).

We used Maxent software (Phillips & Dudík, 2008) to calculate the SDMs. Maxent is a presence‐only modeling software that calculates habitat distributions with a maximum entropy algorithm. We selected the three most relevant climatic variables, based on a preliminary Jack‐knife and variable importance analyses, ecological knowledge of the species, and after excluding highly correlated variables (*r* > 0.7 or VIF >3), to reduce overfitting or collinearity problems. For each model, we randomly split the dataset into 75% (training) and 25% (test) subdatasets (Fielding & Bell, 1997). The final model estimates are averages after 10 repetitions with randomly split subdatasets. To check the effect of variable selection on model performance, we calculated differences in the area under the curve metric (AUC) between the saturated (with all 19 climatic variables) and the minimal model (three variables) for each species. We used 70% of maximum Maxent suitability as a threshold to define habitat suitability.

### 2.2 Dendrochronological methods

In our second analysis, we explored the long‐term growth trend of *H. helix* individuals and their response to increasing carbon availability and changing climate in an Italian case study. We sampled tree rings in *H. helix* and *Q. cerris* in temperate submontane forests in Abruzzo region, central Italy (Supporting Information Figure S1), close to the *Riserva Naturale Guidata Abetina di Rosello*. For a detailed vegetation and fauna description, see Pirone et al. (2005). We targeted *H. helix* rather than other liana species because it is known to form distinctive and regular tree rings that have been cross‐dated successfully in the literature, and it is long‐lived (in our study area, the oldest individual had 79 dated years). It is also important that in our study area, *H. helix* allocates all its growth to one principal stem rather than dividing it between several small ones, thus providing a better proxy for the total biomass of the individual.

In August 2014, we sampled two increment cores per individual from 30 randomly spaced trees and 32 lianas dispersed over the whole study area (Supporting Information Figure S1a). We selected dominant trees to maximize the climatic signal (Schweingruber, 1966) and for similar reasons, avoided sampling the trees on which our sampled lianas were growing to prevent confounding effects of lianas on their hosts. We targeted *Q. cerris* in patches where it dominates the canopy in order to avoid effects of interspecific competition. *Hedera helix* individuals growing as a continuous grass‐like layer were not sampled to avoid pseudoreplication problems. Sampling, cross‐dating, and measurement followed standard dendrochronological methods (Bräker, 2002; Schweingruber, 1966; Speer, 2010). Despite challenging ring recognition in some *H. helix* (Supporting Information Figure S1b,c), tree‐to‐tree agreement in growth patterns allowed accurate cross‐dating of both species (Supporting Information Figure S2a, Table S2). A small percentage of cores with growth anomalies or undetectable rings were discarded, but at least one core was maintained for all but one individual. The tree‐ring data are available online in the International Tree Ring Data Bank depository (accessible at: https://www.ncdc.noaa.gov/data-access/paleoclimatology-data/datasets/tree-ring with access number ITAL045 and ITAL046 for *Q*. *cerris* and *H. helix,* respectively).

We used long‐term climate records from the Climate Research Unit (CRU), University of East Anglia (available at: http://climexp.knmi.nl/) and atmospheric [CO_2_] records from the longest recording measurement, Mauna Loa station (available at: http://www.esrl.noaa.gov/). Each individual tree ring series was transformed to basal area increment (BAI) before analyzing. BAI provides a better quantification of biomass production in ever‐increasing individuals (Schweingruber, 1966):

$${\text{BAI}}_{i}=\pi R_{i}^{2}-\pi R_{i-1}^{2}$$

where *R*_*i*_ represents the radius of the tree in a given year, *i*. We used linear mixed models to model the response of BAI to annual changes in precipitation, temperature, age, and [CO_2_]. Tree identity and series temporal autocorrelation were included as random terms in the model. We included all two‐way interactions between the variables, and BAI was square rooted to meet normality and heteroscedasticity assumptions. We did model selection by backward deletion, based on likelihood ratio tests and comparisons between models using ANOVA and Akaike's information criterion (AIC), to find the most plausible, minimal, model (Crawley, 2013). It is important to notice that in the case of lianas, BAI may underestimate the annual increase in biomass, as lianas are characterized by a stronger increase in length, rather than in diameter, meaning that our results may underestimate liana response to increased carbon availability.

To visualize the interaction between [CO_2_] and maximum temperature on secondary growth for both species, we calculated the change in slope in the growth‐[CO_2_] correlation with regularly increasing maximum temperature (Figure 3). We also showed the overall influence of this interaction on the growth time series for each species by plotting predicted growth over time under‐recorded [CO_2_] and constant [CO_2_]. When assumed to be constant, [CO_2_] was assigned the average value of the complete available record. Tree age was also considered constant in the model predictions (Figure 4).

To reinforce our findings and better understand the observed growth trends, we complemented the tree ring modeling with an assessment of the xylem cavitation risk (Supporting Information Figure S3). In vascular plants, cavitation refers to the breakage of the water transport column by the entrance of air bubbles into the xylem due to strong water tension inside the conduits (Tyree & Zimmermann, 2002). We estimated the vulnerability to cavitation for each tree ring using anatomical measurements of stems from a subsample of individuals of both species (Lens et al., 2011). We collected 20‐μm slides of seven *H. helix* and 11 *Q. cerris,* stained them with 1% safranin–astra blue (Arbellay, Fonti, & Stoffel, 2012), and analyzed them using WinCell Pro V 2004a to obtain a time series of vulnerability per tree. We chose the oldest *H. helix* individuals and the youngest *Q. cerris* individuals to minimize age differences between species. The xylem vulnerability index (VI) was calculated as $\text{VI}=D_{v}\cdot V_{f}^{-1}$, where the circular vessel diameter (*D*_v_) is calculated following White's equation, and *V*_f_ is the total number of earlywood vessels counted per unit (Arbellay et al., 2012; Carlquist, 2001; Lens et al., 2011).

### 2.3 Effects of carbon fertilization on *H. helix* model predictions

To illustrate how a positive effect of increasing [CO_2_] could affect model predictions of future habitat suitability, we defined three potential CO_2_‐effect scenarios for future *H. helix* habitat distributions (Figure 4). These were as follows: “no fertilization,” “slight carbon fertilization,” (simply assuming a 10% increase in habitat suitability across Europe following carbon fertilization), and “strong carbon fertilization” (a 20% increase in habitat suitability) scenarios. These numbers are necessarily arbitrary and used for explorative purposes only, as there is no available method to translate growth responses into changes in habitat suitability. They are also different to the previously described modeling approach that reconstructs growth with and without CO_2_ effects. A 10%–20% increase in habitat suitability seems a realistic assumption given the strong response to [CO_2_] observed in our long time series, as well as the strong, unsaturated response of *H. helix* to carbon fertilization in previous experimental studies. Hättenschwiler and Körner (2003) recorded up to 100% increase in biomass and 137% in stem length under 660 ppm of CO_2_, without signs of carbon saturation, and Zotz et al. (2006) similarly observed a 30%–60% increase in length and biomass increment in shoots of *H. helix* under elevated CO2. All these analyses were carried out in the R software (R Core Team 2015).

## 3 RESULTS AND DISCUSSION

### 3.1 Climate‐based spatial distribution models

**Figure 2:**
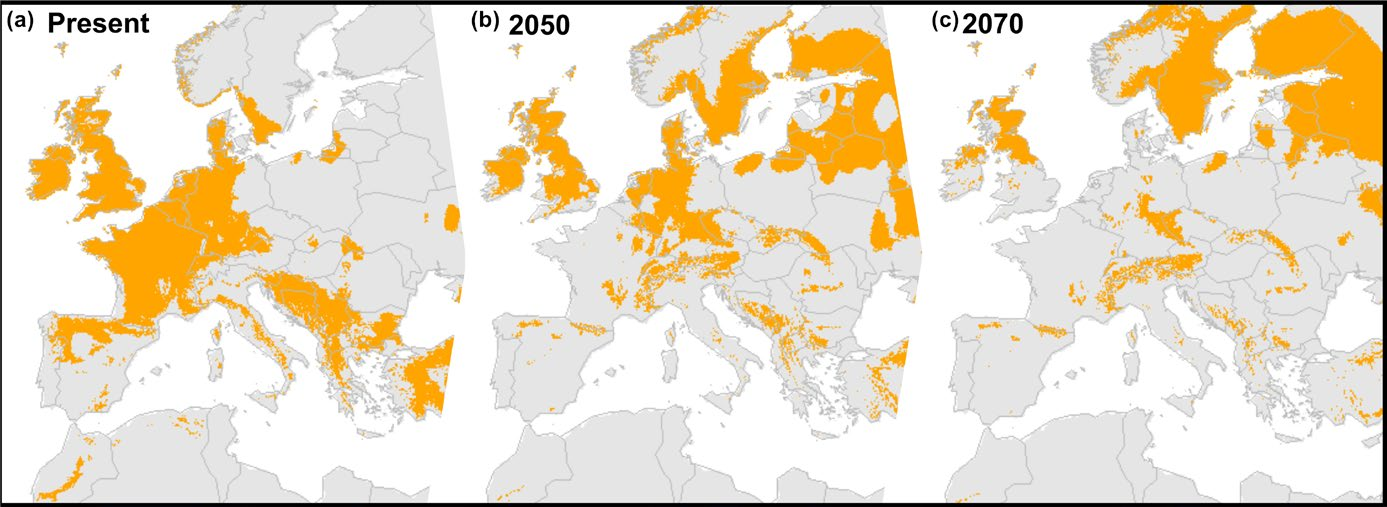
Climate‐only models for the suitable habitat of *Hedera helix* predict slight changes in the total habitat area but large range shifts toward north and east in future projections. (a–c) Species distribution models show the change in area suitable for *H. helix* from the present to 2050 and 2070

The SDMs showed that changes in temperature and precipitation alone will likely alter the amount of habitat suitable for *H. helix* in Europe, requiring large range shifts for the species to track its climatic niche (Figure 2). The *H. helix* climatic niche was mainly defined by precipitation in the coldest quarter of the year (51% permutated variable importance), annual mean temperature (42.6% variable importance), and precipitation in the wettest quarter (6.4% variable importance). The positive correlation with mean temperature is in line with the biogeographical history of *H. helix*, which evolved from tropical families under warmer climates (Metcalfe, 2005). Our models project a slight increase in *H. helix* habitat by 2050 and a slight decrease by 2070 (Figure 2), but there is a large shift in the species range toward the north and east in the future (Figure 2). A future increase in the mean temperature in eastern and northern parts of Europe might make these regions suitable for frost‐sensitive liana species in the near future (Gianoli, 2015; Schnitzer, 2005). Also, a positive correlation with winter precipitation supports the hypothesis that evergreen lianas may profit more from warmer conditions in winter and early spring than their broadleaved counterparts, as they can benefit from high light conditions before tree bud break occurs (Schnitzer, 2005). In central and southern Europe, increasingly dry conditions are expected to translate into a decrease in the amount of suitable habitat, with *H. helix* expected to be retained only in mountainous areas (Figure 2b,c). Changes in temperature and precipitation could therefore substantially alter southern European forests by causing the loss of *H. helix*.

Our model was robust to variable selection, as the reduction in AUC values between the saturated (19 climatic variables) and minimal (three climatic variables) models was lower than 0.1% (AUC: 0.771; AUC_s_ = 0.774). The AUC values were slightly smaller than the usual 0.8 threshold considered for model evaluation, which may be caused by high species plasticity or intraspecific variability. As in all SDMs, habitat suitability models do not necessarily represent the future realized distribution of the species. The actual responses of populations to environmental change are more complex and include factors such as dispersion, microenvironmental conditions, human disturbances, host preference, and other biotic environmental factors, which interact with habitat suitability to define the realized presence of the species (Fitzpatrick & Keller, 2015). A particularly relevant factor to define the realized distribution in the case of lianas is host preference. However, it is unlikely that this factor plays an important role in the distribution of *H. helix*, as it seems to have low host specificity and preferences are driven more by size and bark characteristics (Castagneri et al., 2013) or forest successional stage (Ladwig & Meiners, 2010) than responses to particular tree species.

### 3.2 Long‐term climate interactions using tree rings

**Figure 3:**
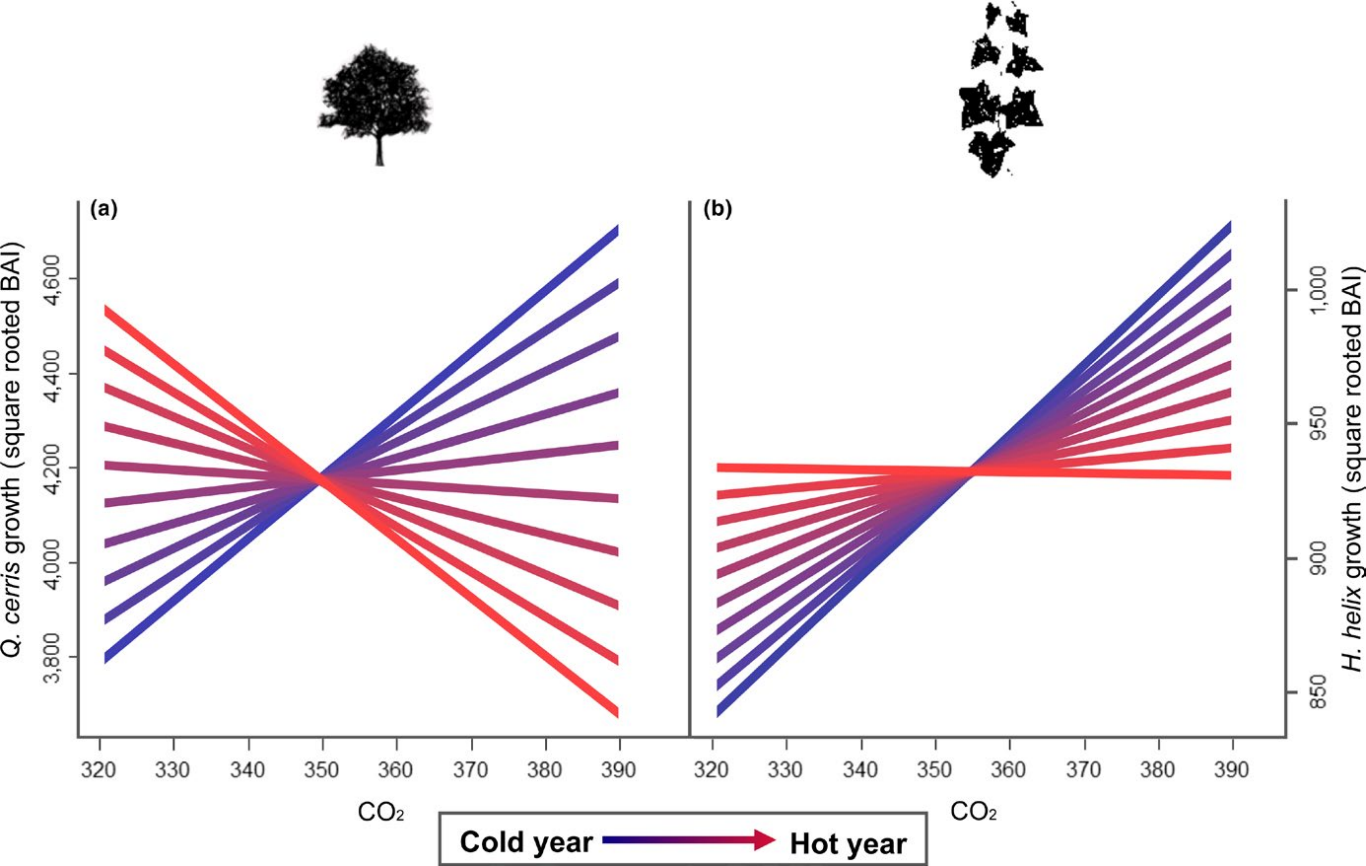
Effect of CO_2_ and temperature on the growth of a liana and its host. (a) *Quercus cerris* growth (measured as squared rooted basal area increment) showed a strong interactive response to atmospheric CO_2_ concentration and temperature, where CO_2_ increased growth in cool years but decreased it in hot years. (b) By contrast, *Hedera helix* grew better with increased CO_2_ under almost all temperature conditions. Predictions are shown for temperatures within the 95% quantile range

**Figure 4:**
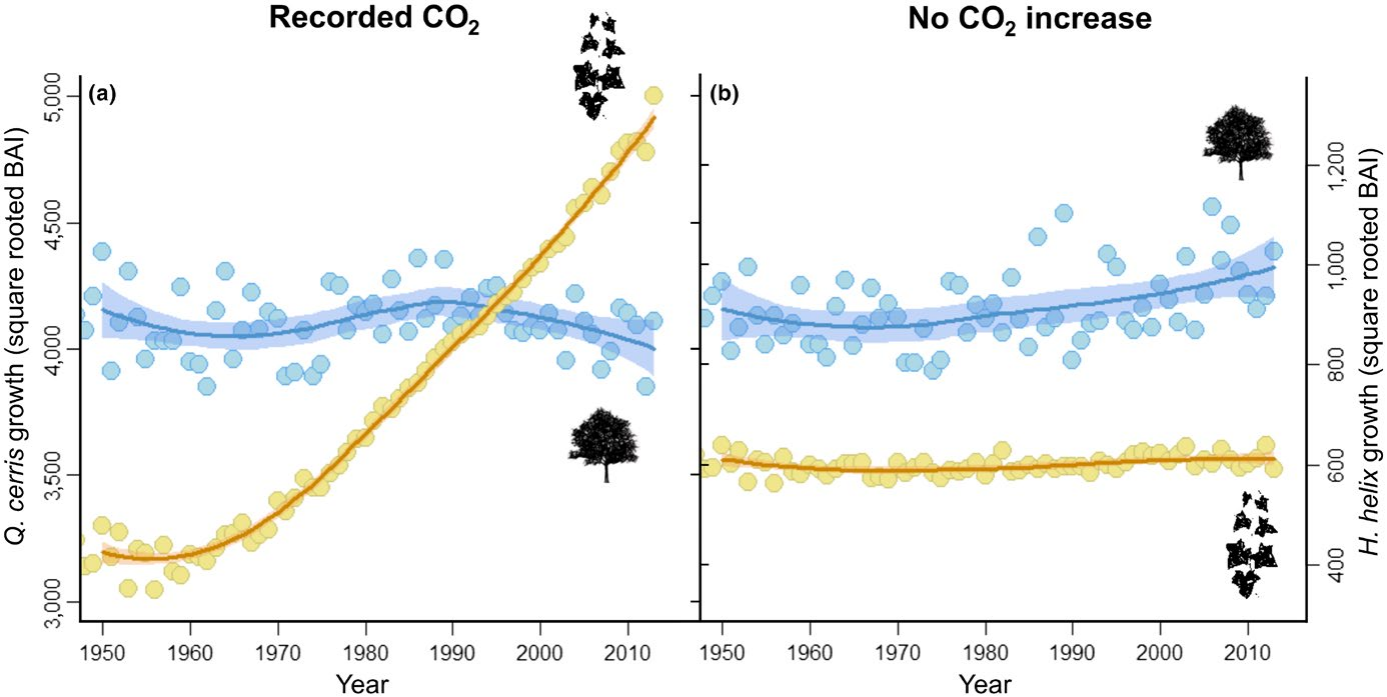
Effect of CO_2_ on growth trend in *Hedera helix* and *Quercus cerris* growth during the period 1950–2013. (a) Growth reconstruction (measured as square‐root transformed basal area increment) for the time series of *H. helix* (orange) and *Q. cerris* (blue) using model predictions for the measured CO_2_, temperature and precipitation conditions in each year. (b) Reconstruction assuming constant CO_2_ concentration but the measured values of temperature and precipitation in each year. To remove age effects on growth, the age of each species is set to the species average. Note that the growth of the two species is displayed on a different scale

While our models predict that changes in temperature and precipitation are likely to impact *H. helix* distribution, they do not account for the effects of increasing [CO_2_]. The tree ring analysis, on the other hand, showed this is an important factor, as we found a strong and significant correlation between increasing [CO_2_] and growth for both *H. helix* and *Q. cerris*. This is consistent with experimental results (Körner, Morgan, & Richard Norby, 2007; Zotz et al., 2006). Importantly, however, the effects of carbon fertilization depended on temperature ([CO_2_] × maximum temperature interaction) and the strength and shape of this interaction differed between the liana and the tree (Figure 3). *Q. cerris* trees only grew better with increasing [CO_2_] under cool conditions, while their growth was reduced by increasing [CO_2_] under warm conditions. By contrast, *H. helix* benefited from increasing [CO_2_] under most recorded climatic conditions, although the positive effect of carbon fertilization decreased in warmer conditions. The model predictions assuming constant or observed [CO_2_] revealed that the increase in *H. helix* growth since the 1950s could not be explained by temperature alone, but was correlated with increasing [CO_2_] (Figure 4, Supporting Information Figure S3a,b). *Q. cerris* growth, on the other hand, has not increased over time. The trends in vulnerability to cavitation support these findings: Increasing cavitation risk with increasing temperature suggests that the inability of *Q. cerris* to respond to increasing [CO_2_] concentrations is likely related to its greater sensitivity to drought (Supporting Information Figure S3c,d). The increased growth of *H. helix*, on the other hand, is not accompanied by an increase in vulnerability to cavitation, further suggesting that *H. helix* seems to take greater advantage of increasing [CO_2_] than its tree host, as tree growth seemed to have been limited by the simultaneous rise in temperature and water stress.

In recent years, there has been a large controversy about whether tree ring measurements can be used for evaluating historical growth patterns (Bowman, et al. 2012; Brienen, Gloor, & Ziv, 2016; Brienen, Gloor, & Zuidema, 2012) because of the so‐called big‐tree selection bias and slow‐grower survivorship bias that may confound climate growth trends with population dynamics or sampling strategies (Brienen et al., 2012). A central aspect of both biases is the unbalanced relationship between age and annual growth in the sampled population, resulting in a spurious positive trend in growth produced by the different composition of slow‐ and fast‐growing trees in each sampled age class (Brienen et al., 2016; more info in Supporting Information). We found no significant correlation between age and mean annual growth in our samples (Supporting Information Figure S4). Therefore, we do not expect these biases to influence our results. We also do not expect substantial disturbance or logging to have occurred in our study area, which has been protected since 1997. Although we cannot completely rule out the possibility that our forests are recovering from some old disturbance, the well‐distributed samples across the landscape and their consistency make this unlikely.

### 3.3 Potential consequences for the spatial distribution models

**Figure 5:**
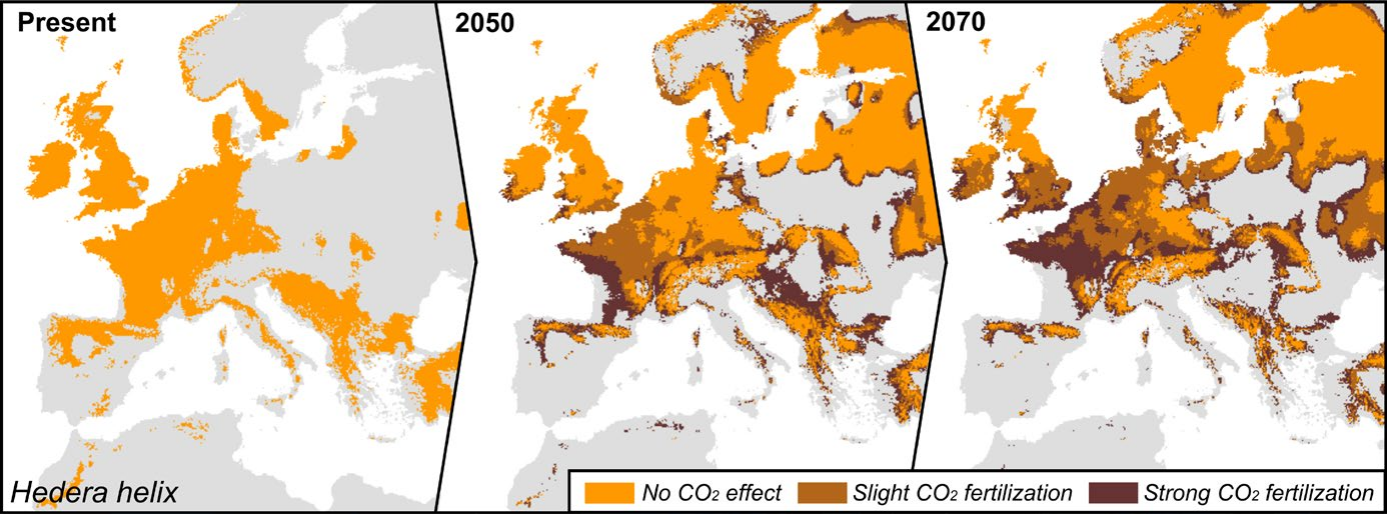
Increased CO_2_ may allow a liana to persist in otherwise unsuitable areas. Suitable habitat area for *Hedera helix* without carbon fertilization effect (orange); the extra habitat that would be suitable with a slight carbon fertilization effect (dark orange: 10% increase in habitat suitability in the model), and extra habitat that would be suitable assuming strong carbon fertilization (brown: 20% increase in habitat suitability in the model)

We illustrate how the positive effects of [CO_2_] on *H. helix* might affect its future distribution, using three carbon fertilization scenarios. Even small carbon fertilization effects could drastically change the predictions of how *H. helix* will cope with drier conditions in central and southern Europe, as well the extent to which it will expand northwards (Figure 5). It is noteworthy that, due to the nature of the interaction between the temperature and carbon fertilization effects, it is likely that our model underestimates suitability in the colder areas of Europe while overestimating it for the hotter southern European localities, as [CO_2_] effects were found to be lower at high temperature (Supporting Information Figure S5). Whether the increase in growth that we observed over time in response to [CO_2_] would translate into changes in range size remains speculative. Our results stress the need for methodological development to mechanistically include the effects of [CO_2_] and their interactions in SDMs. Our findings seem to indicate that CO_2_ fertilization effects and their interactions can have important consequences for ecological modeling and thus cannot be ignored when modeling future species distributions.

Liana species other than *H. helix* may not respond similarly to increased [CO_2_]. Deciduous species, in particular, may not profit as strongly as evergreen ones because they do not benefit from the increase in the growing season more than their host trees (Schnitzer, 2005). To test the effect of carbon fertilization on other liana species, [CO_2_] fertilization experiments coupled with dendrochronological studies, where possible, would be required. It is suggested that under high long‐term [CO_2_], other resources (e.g. soil phosphorus) can become limiting to plant growth, and therefore, no long‐term fertilization effect would be expected (Bader et al., 2013). We found no saturating effect in the carbon fertilization curve; however, as our approach is not manipulative, carbon fertilization effects are limited to those increases which have occurred in the last decades. The growth response that we observed is therefore in response to much lower [CO_2_] than those used in fertilization experiments but it is in line with reports from other fertilization experiments with temperate lianas that did not find a reduction in carbon fertilization effect after a 80% increase in [CO_2_]. This is a much larger increase than what has been observed in the last decades (Hättenschwiler & Körner, 2003). However, saturating effects could appear in the future as [CO_2_] increases further. These potentially complex effects further underline the need to consider interactions between factors when predicting responses to climate change.

## 4 CONCLUSIONS

We found that climate‐only models predicted slight changes in the total area suitable for *Hedera helix* in the future, accompanied by large shifts in the species range toward the north and east of Europe. These models, however, could not account for the effects related to an increase in [CO_2_] or the interactions between climate change variables, which our long‐term case study suggests can have a significant effect on liana growth. Our models show that even small increases in habitat suitability, related to carbon fertilization, might substantially influence the future distribution of *H. helix*, allowing it to expand and persist in areas where it would not otherwise. Our results therefore highlight the need for new approaches to distribution modeling, not only to account for carbon fertilization as we showed here, but also to include its interactions with changing climate. Long‐term growth observations and experiments assessing the response of different species to changing climatic conditions are mostly lacking, and the combination of multiple approaches, such as tree ring analysis and classical modeling employed here, can complement traditional manipulative experiments and further improve the accuracy of ecological models.

## AUTHOR CONTRIBUTIONS

RDM conceived and led the project. RDM, JBC, and FS designed the experimental sampling and performed the dendrochronological and ecophysiological analyses. RDM and EA performed the statistical analyses. All authors contributed to data interpretation and writing in consecutive versions of the manuscript.

## DATA ACCESSIBILITY

Presence data: publicly available data from Global Biodiversity Information Facility (GBIF, http://www.gbif.org). Environmental data: publicly available data from http://climexp.knmi.nl/and http://www.esrl.noaa.gov/. Tree Ring Data: have been submitted to the International Tree Ring Data Bank and publicly available at: http://www.ncdc.noaa.gov/data-access/paleoclimatology-data/datasets/tree with access number ITAL045 and ITAL046 for *Q*. *cerris* and *H. helix* respectively. Cavitation data available from the Dryad Digital Repository: https://doi.org/10.5061/dryad.5688g40.

## References

[ece34388-bib-0001] Allen, B. P. , Sharitz, R. R. , & Goebel, P. C. (2007). Are lianas increasing in importance in temperate floodplain forests in the southeastern United States? Forest Ecology and Management, 242, 17–23. 10.1016/j.foreco.2007.01.027

[ece34388-bib-0002] Arbellay, E. , Fonti, P. , & Stoffel, M. (2012). Duration and extension of anatomical changes in wood structure after cambial injury. Journal of Experimental Botany, 63, 3271–3277. 10.1093/jxb/ers050 22378953

[ece34388-bib-0003] Bader, M. K.‐F. , Leuzinger, S. , Keel, S. G. , Siegwolf, R. T. W. , Hagedorn, F. , Schleppi, P. , & Körner, C. (2013). Central European hardwood trees in a high‐CO_2_ future: Synthesis of an 8‐year forest canopy CO_2_ enrichment project. Journal of Ecology, 101, 1509–1519. 10.1111/1365-2745.12149

[ece34388-bib-0004] Bowman, D. M. J. S. , Brienen, R. J. W. , Gloor, E. , Phillips, O. L. , & Prior, L. D. (2012). Detecting trends in tree growth: Not so simple. Trends in Plant Science, 18, 11–17. 22960000 10.1016/j.tplants.2012.08.005

[ece34388-bib-0005] Bräker, O. U. (2002). Measuring and data processing in tree ring research – a methodological introduction. Dendrochronologia, 20(1‐2), 203–216. 10.1078/1125-7865-00017

[ece34388-bib-0006] Brienen, R. J. W. , Gloor, M. , & Ziv, G. (2016). Tree demography dominates long‐term growth trends inferred from tree rings. Global Change Biology, 23, 474–484. 27387088 10.1111/gcb.13410 PMC6849721

[ece34388-bib-0007] Brienen, R. J. W. , Gloor, E. , & Zuidema, P. A. (2012). Detecting evidence for CO_2_ fertilization from tree ring studies: The potential role of sampling biases. Global Biogeochemical Cycles, 26, GB1025, 10.1029/2011gb004143

[ece34388-bib-0008] Carlquist, S. (2001) Comparative wood anatomy. Systematic, ecological, and evolutionary aspects of dicotyledon wood. Heidelberg, Germany: Springer Berlin Heidelberg.

[ece34388-bib-0009] Castagneri, D. , Garbarino, M. , & Nola, P. (2013). Host preference and growth patterns of ivy (*Hedera helix* L.) in a temperate alluvial forest. Plant Ecology, 214, 1–9. 10.1007/s11258-012-0130-5

[ece34388-bib-0010] Crawley, M. J. (2013). The R book, 2nd ed. Imperial College London at Silwood Park, UK. Chichester, UK: John Wiley & Sons Ltd.

[ece34388-bib-0011] Fielding, A. H. , & Bell, J. F. (1997). A review of methods for the assessment of prediction errors in conservation presence/absence models. Environmental Conservation, 24, 38–49. 10.1017/S0376892997000088

[ece34388-bib-0012] Fitzpatrick, M. C. , & Keller, S. R. (2015). Ecological genomics meets community‐level modelling of biodiversity: Mapping the genomic landscape of current and future environmental adaptation. Ecology Letters, 18, 1–16. 10.1111/ele.12376 25270536

[ece34388-bib-0013] Garfi, G. , & Ficarrotta, S. (2003). Influence of ivy (*Hedera helix* L.) on the growth of downy oak (*Quercus pubescens* s.l.) in the Manti Carcaci Nature Reserve (central‐western Sicily). Ecologia Mediterranea, 29, 5–14.

[ece34388-bib-0014] Gianoli, E. (2015). Evolutionary implications of the climbing habit in plants In Schnitzer S., Bongers F., Burnham R. J. & Putz F. E. (Eds.), Ecology of lianas (pp. 239–250). Chichester, UK: John Wiley & Sons, Ltd.

[ece34388-bib-0015] Hättenschwiler, S. , & Körner, C. (2003). Does elevated CO_2_ facilitate naturalization of the non‐indigenous *Prunus laurocerasus* in Swiss temperate forests? Functional Ecology, 17, 778–785. 10.1111/j.1365-2435.2003.00785.x

[ece34388-bib-0016] Heinrichs, S. , & Schmidt, W. (2015). Dynamics of *Hedera helix* L. in Central European beech forests on limestone: Results from long‐term monitoring and experimental studies. Plant Ecology, 216, 1–15. 10.1007/s11258-014-0412-1

[ece34388-bib-0017] Heuzé, P. , Dupouey, J.‐L. , & Schnitzler, A. (2008). Radial growth response of *Hedera helix* to hydrological changes and climatic variability in the Rhine floodplain. River Research and Applications, 25, 393–404.

[ece34388-bib-0018] Hijmans, R. J. , Cameron, S. E. , Parra, J. L. , Jones, P. G. , & Jarvis, A. (2005). Very high resolution interpolated climate surfaces for global land areas. International Journal of Climatology, 25, 1965–1978. 10.1002/(ISSN)1097-0088

[ece34388-bib-0019] Körner, C. (2004). Through enhanced tree dynamics carbon dioxide enrichment may cause tropical forests to lose carbon. Philosophical Transactions of the Royal Society B: Biological Sciences, 359, 493–498. 10.1098/rstb.2003.1429 PMC1693332 15212098

[ece34388-bib-0020] Körner, C. , Morgan, J. , & Richard Norby, R. (2007). CO_2_ fertilization: When, where, how much? In Canadell J. G., Pataki D. E., & Pitelka L. F. (Eds.), Terrestrial ecosystems in a changing World: Global Change (pp. 9–21). The IGBP series. Berlin, Germany: Springer 10.1007/978-3-540-32730-1

[ece34388-bib-0021] Ladwig, L. M. , & Meiners, S. J. (2010). Spatiotemporal dynamics of lianas during 50 years of succession to temperate forest. Ecology, 91, 671–680. 10.1890/08-1738.1 20426327

[ece34388-bib-0022] Ledo, A. , & Schnitzer, S. A. (2014). Disturbance and clonal reproduction determine liana distribution and maintain liana diversity in a tropical forest. Ecology, 95, 2169–2178. 10.1890/13-1775.1 25230468

[ece34388-bib-0023] Lens, F. , Sperry, J. S. , Christman, M. A. , Choat, B. , Rabaey, D. , & Jansen, S. (2011). Testing hypotheses that link wood anatomy to cavitation resistance and hydraulic conductivity in the genus *Acer* . New Phytologist, 190, 709–723. 10.1111/j.1469-8137.2010.03518.x 21054413

[ece34388-bib-0024] Lindner, M. , Fitzgerald, J. B. , Zimmermann, N. E. , Reyer, C. , Delzon, S. , van der Maaten, E. , … Hanewinkel, M. (2014). Climate change and European forests: What do we know, what are the uncertainties, and what are the implications for forest management? Journal of Environmental Management, 146, 69–83. 10.1016/j.jenvman.2014.07.030 25156267

[ece34388-bib-0025] Martin, G. M. , Bellouin, N. , Collins, W. J. , Culverwell, I. D. , Halloran, P. R. , Hardiman, S. C. , … Wiltshire, A. (2011). The HadGEM2 family of Met Office Unified Model climate configurations. Geoscientific Model Development, 4, 723–757.

[ece34388-bib-0026] Marvin, D. C. , Winter, K. , Burnham, R. J. , & Schnitzer, S. A. (2015). No evidence that elevated CO_2_ gives tropical lianas an advantage over tropical trees. Global Change Biology, 21, 2055–2069. 10.1111/gcb.12820 25471795

[ece34388-bib-0027] Metcalfe, D. J. (2005). *Hedera helix* L. Journal of Ecology, 93, 632–648. 10.1111/j.1365-2745.2005.01021.x

[ece34388-bib-0501] Nola, P. (1997). Interactions between *Fagus sylvatica* L. and *Hedera helix* L.: A dendroecological approach. Dendrochronologia, 15, 23–37.

[ece34388-bib-0028] Norby, R. J. , & Luo, Y. (2004). Evaluating ecosystem responses to rising atmospheric CO_2_ and global warming in a multi‐factor world. New Phytologist, 162, 281–293. 10.1111/j.1469-8137.2004.01047.x

[ece34388-bib-0029] Phillips, S. J. , & Dudík, M. (2008). Modeling of species distributions with Maxent: New extensions and a comprehensive evaluation. Ecography, 31, 161–175. 10.1111/j.0906-7590.2008.5203.x

[ece34388-bib-0030] Phillips, O. L. , Vásquez Martínez, R. , Arroyo, L. , Baker, T. R. , Killeen, T. , Lewis, S. L. , … Vinceti, B. (2002). Increasing dominance of large lianas in Amazonian forests. Nature, 418, 770–774. 10.1038/nature00926 12181565

[ece34388-bib-0031] Pirone, G. , Ciaschetti, G. , & Frattaroli, A. R. (2005). La vegetazione della Riserva Naturale Regionale “Abetina di Rosello” (Abruzzo, Italia). Fitosolociologia, 42, 121–137.

[ece34388-bib-0032] R Core Team . (2015). R: A language and environment for statistical computing. Vienna, Austria: R Foundation for Statistical Computing.

[ece34388-bib-0033] Schnitzer, S. A. (2005). A mechanistic explanation for global patterns of liana abundance and distribution. American Naturalist, 166, 262–276. 10.1086/431250 16032578

[ece34388-bib-0034] Schnitzer, S. A. (2015). Increasing liana abundance in neotropical forests: Causes and consequences In Schnitzer S. A., Bongers F., Burnham R. J., & Putz F. E. (Eds.), Ecology of lianas (pp. 461–464). Chichester, UK: John Wiley & Sons Ltd.

[ece34388-bib-0035] Schnitzer, S. A. , & Bongers, F. (2002). The ecology of lianas and their role in forests. Trends in Ecology & Evolution, 17, 223–230. 10.1016/S0169-5347(02)02491-6

[ece34388-bib-0036] Schnitzer, S. A. , & Bongers, F. (2011). Increasing liana abundance and biomass in tropical forests: Emerging patterns and putative mechanisms. Ecology Letters, 14, 397–406. 10.1111/j.1461-0248.2011.01590.x 21314879

[ece34388-bib-0037] Schweingruber, F. H. (1966). Tree rings and environment dendroecology. Berne, Switzerland: Paul Haupt.

[ece34388-bib-0038] Silvertown, J. W. (2008). Demons in Eden. The paradox of plant diversity. Chicago, IL: University of Chicago Press.

[ece34388-bib-0039] Speer, J. H. (2010). Fundamentals of tree ring research. Tucson, AZ: University of Arizona Press.

[ece34388-bib-0040] Tymen, B. , Réjou‐Méchain, M. , Dalling, J. W. , Fauset, S. , Feldpausch, T. R. , Norden, N. , … Chave, J. (2016). Evidence for arrested succession in a liana‐infested Amazonian forest. Journal of Ecology, 104, 149–159. 10.1111/1365-2745.12504

[ece34388-bib-0041] Tyree, M. T. , & Zimmermann, M. H. (2002). Xylem structure and the ascent of sap. Heidelberg, Germany: Springer Heidelberg 10.1007/978-3-662-04931-0

[ece34388-bib-0042] van der Heijden, G. M. , Powers, J. S. , & Schnitzer, S. A. (2015). Lianas reduce carbon accumulation and storage in tropical forests. Proceedings of the National Academy of Sciences of the United States of America, 112(43), 13267–13271. 10.1073/pnas.1504869112 26460031 PMC4629347

[ece34388-bib-0043] Zotz, G. , Cueni, N. , & Körner, C. (2006). In situ growth stimulation of a temperate zone liana (*Hedera helix*) in elevated CO_2_ . Functional Ecology, 20, 763–769. 10.1111/j.1365-2435.2006.01156.x

