# Supplementary material for: Increase in CO 2 concentration could alter the response of Hedera helix to climate change
Source: Ecol Evol. 2018 Jul 30;8(16):8598–606. doi: 10.1002/ece3.4388 (PMC6144985; doi:10.1002/ece3.4388)
Supplement: Supplementary file 1 [file ECE3-8-8598-s001.docx]

**Supplementary material and methods**

*Species distribution modelling*

We calculated *Hedera helix* Species Distribution Models (SDMs) using Maxent software (Phillips and Dudik 2008) and raster of climatic variables (Fig. S1). The full model included 19 climatic variables in the Bioclim dataset (Hijmans *et al.* 2005). The bioclim variables are widely used in correlational species distribution models to quantify the environmental niche of species (Booth *et al.* 2013). Bioclim variables were downloaded from <http://www.worldclim.org/bioclim> and include: mean annual temperature and precipitation (2 variables); mean diurnal range (1); isothermality (1); temperature and precipitation seasonality (2); maximum temperature of the warmest month (1); minimum temperature of the coldest month (1); temperature annual range (1); mean temperature and precipitation of the wettest, driest, warmest, and coldest quarters (8); and precipitation of the wettest and driest months (2). We selected the 3 most influential variables based in permutated importance using a saturated model including all variables.

*Dendrochronological sampling and study area*


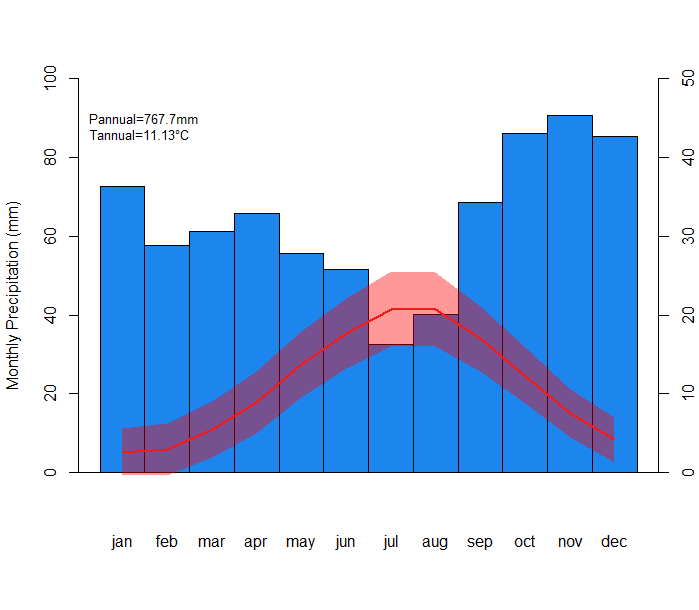

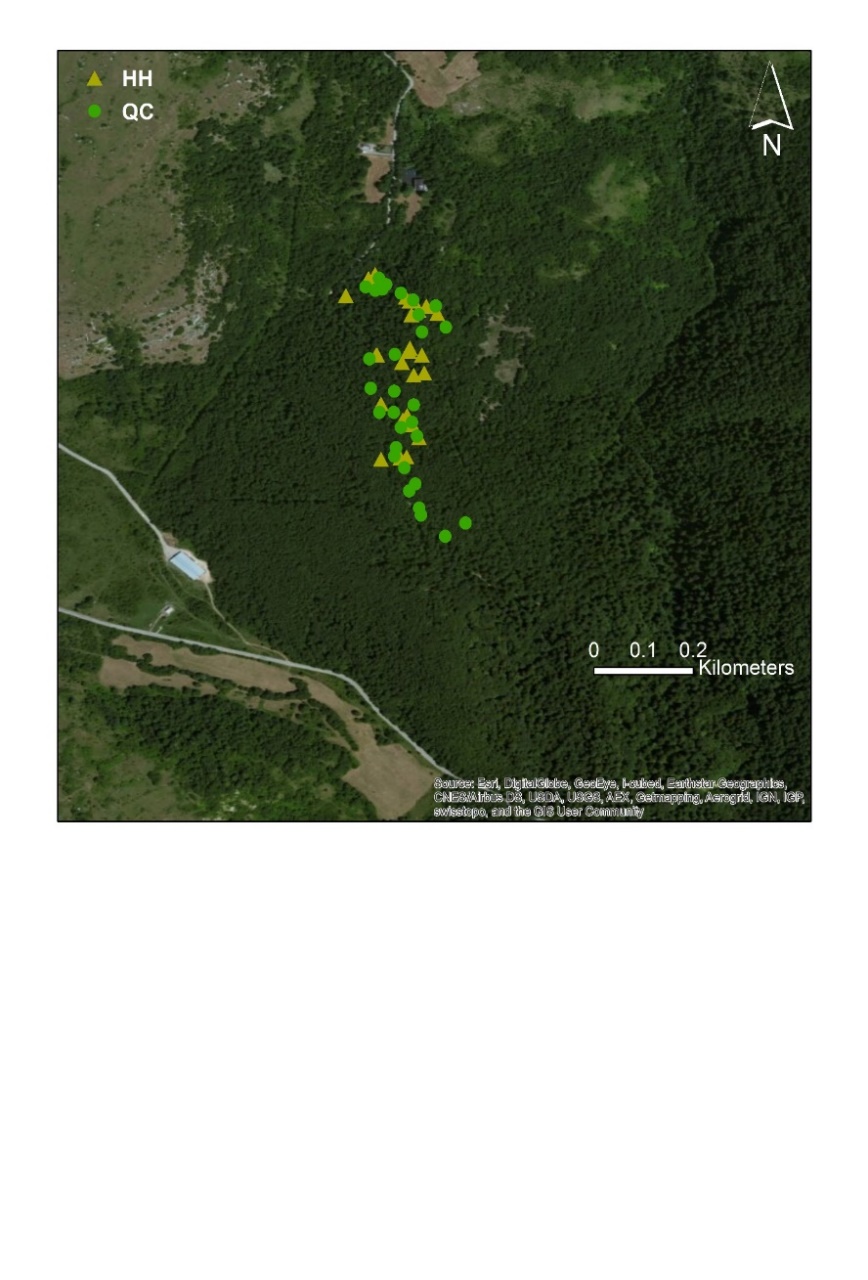

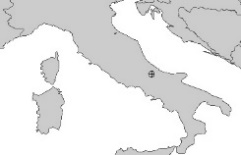


**a**

**b**

**Fig. S1 a,** location of the study site and the sampled *Quercus cerris* (green circles, QC), and *Hedera helix* (yellow triangles, HH) individuals. **b,** climatic diagram of the study area presenting precipitation (blue bars) and temperature (red line) century-average values. The red-shaded area shows maximum and minimum monthly temperatures

We took dendrochronological samples from old growth *Quercus cerris* (*Q. cerris*) and *H. helix* individuals from a *Quercus-Abies* dominated forest located in the central region of Abruzzo, Italy (41°53’N 14°21’E) (Fig. S1a). The study area is located at 900-1000 m above sea level, close to the *Riserva Natural Guidata Abetina di Rosello*. The climate is temperate sub-montane with an annual precipitation of 768 mm, distributed throughout the year but peaking in spring and autumn. Average annual temperature is 11.13 °C (Fig. S1b). Soils in the study area are mostly calcareous marl and clays. The surrounding forests are mostly dominated by *Abies alba*, mixed with *Fagus sylvatica*, *Quercus cerris*, *Tilia platyphyllos*, and *Fraxinus excelsior* as part of the dominant canopy. Monocultures of *Abies alba, Fagus sylvatica*, and *Quercus cerris* also occur (see Pirone *et al.*, 2005).

We sampled *Q. cerris* and *H. helix* individuals from *Q. cerris* dominated forests to eliminate potential confounding effects related with the effect of canopy diversity on the performance of trees and lianas. Higher tree diversity is known to affect both forest productivity (Liang *et al.* 2016) and its growth stability (Jucker *et al.* 2014). Wood cores were taken at 1.3m (breast height, 1.3m from the last rooting point in the case of liana individuals), 2 cores per individual, using a Pressler borer (Häglof, Langsele, Sweden). Sampling at breast height has the side effect of reducing the number of sampled years, but also it increases the reliability in the detection of growth trends, by reducing the likelihood of wood anomalies that could affect growth assessment. Anomalies such as trunk flares or buttress roots, which are more frequent in the low parts of the trunk (Speer 2010), can strongly alter the detection of growth trends. We targeted dominant, mature individuals, with no signs of illness or damage, separated at least 25m from each other or from permanent waters streams; in order to maximize the climatic signal in their growth (Schweingruber 1966). In the case of *H. helix*, dominance was difficult to estimate without using complex construction cranes (Leuzinger *et al.* 2011). Therefore, we used size as a proxy for dominance, selecting big, healthy-looking, and old individuals. As the main interest of this work is the relationship of both species with climate, rather than the effects of liana on tree growth, we avoided sampling both individuals of the liana-host pair.

**Fig. S2 a,** master chronologies for *Hedera helix* (orange) and *Quercus cerris* (blue). Individual tree ring series are also shown. Vertical dashed lines show pointer years common for both chronologies. Stereoscopic details of *Hedera helix*, **b,** and *Quercus cerris,* **c** rings. Side arrows mark the limit between annual rings


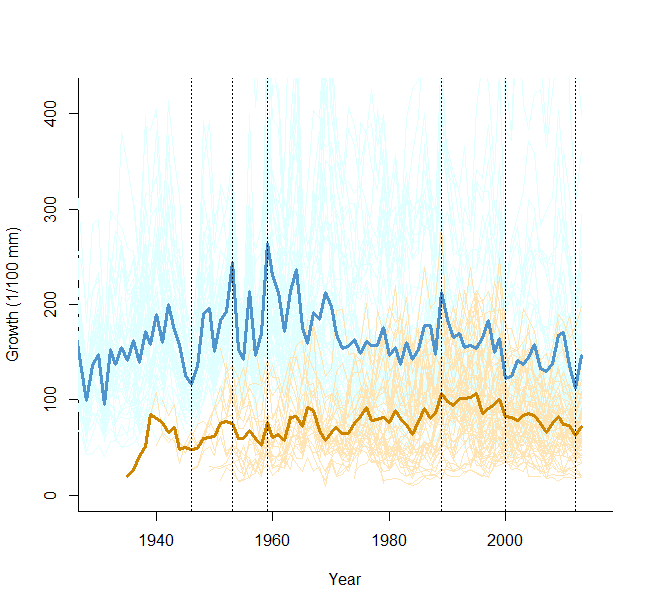

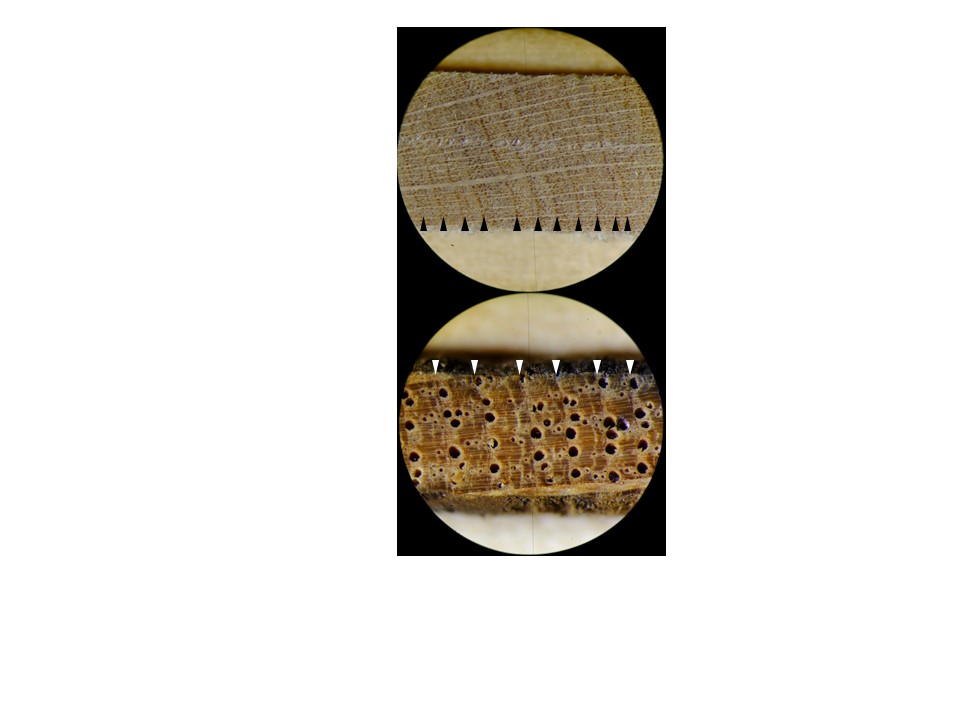


**c**

**b**

**a**

Both species had distinguishable rings that allowed for reliable crossdating, with medium sensitivity and high inter-series correlation (**Table S1**), as well as good visual agreement between species (**Fig. S2a**). Both *H. helix* semi-porous rings (**Fig. S2b**), and *Q. cerris* porous rings (**Fig. S2c**) were easy to identify in most of the cores and rings. Statistical checking and tree ring measurements followed standard dendrochronological methods (Speer 2010).

**Table S1** Statistics of the dendrochronological analysis.

|  | *Quercus cerris* | *Hedera helix* |
| --- | --- | --- |
| Number of trees | 30 | 32 |
| Number of crossdated cores | 65 | 54 |
| Mean tree-ring growth (mm) | 1.72 | 0.81 |
| Mean age | 90 | 43 |
| Maximum crossdated age | 142 | 79 |
| Mean DBH (mm) | 45.6 | 8.20 |
| Mean sensitivity | 0.190 | 0.212 |
| Inter-series correlation | 0.680 | 0.428 |

*Growth modelling*

We modelled growth as a function of climate using linear mixed models (see Pinheiro *et al.* 2016). Tree-ring growth series were first transformed to basal area increment (BAI) series, as this is a better characterization of biomass production in ever-increasing individuals. BAI corrects for the increasing volume of wood produced annually for the same ring-width in individuals with increasingly bigger sizes (Schweingruber 1966).

The individual-based model was defined as follows, in R code:

sqrt(*BAI*) ~ (*P + T + CO_2_ + A*)^2 *+ AC,* random =~1|*Tree*

Where *BAI* is expressed in mm^2^, *P* are precipitation-related variables in mm*month^-1^, *T* are temperature-related variables in °C, *A* is the age of the tree, *Tree* is a random factor that accounts for tree identity, and *AC* is an autocorrelative term to deal with temporal autocorrelation in the BAI series. Interactions between factors were allowed up to the second order. We square root-transformed BAI to correct for normality, heterocedasticity, and the overdispersion of the residuals. Age was calculated as the total number of measured rings when pith was present in the core. In those cores with missing pith, the number of absent rings until the pith were estimated using the pith indicator method (Speer, 2010).

Variable selection was done by backward elimination of interactions and variables and model comparisons by ANOVAs until the minimal model was met (**Table S2**; Crawley 2013). To ensure consistency after the variable selection, we checked that the correlations with the untransformed raw values were in agreement with the modelled parameters for both growth (**Fig. S3a,b**) and cavitation vulnerability (**Fig. S3c,d**, see methods in the main text).

It is important to note that the correlations between mean annual temperature, maximum annual temperature and [CO_2_] were significant, as expected, but R^2^ values were low (R^2^_CO2-MaxTempJune_ = 0.31, R^2^_CO2-MeanAnnualTemp_ = 0.51, and R^2^_MaxTempJune-MeanAnnualTemp_ = 0.31). Since the R^2^ between CO2 and mean annual temperature was slightly above the threshold value, we assessed collinearity using the variance inflation factor (VIF) via the ‘usdm’ package in R, which showed VIF < 3 for all variables. Therefore, no problems of fitting due to high collinearity between variables are expected.


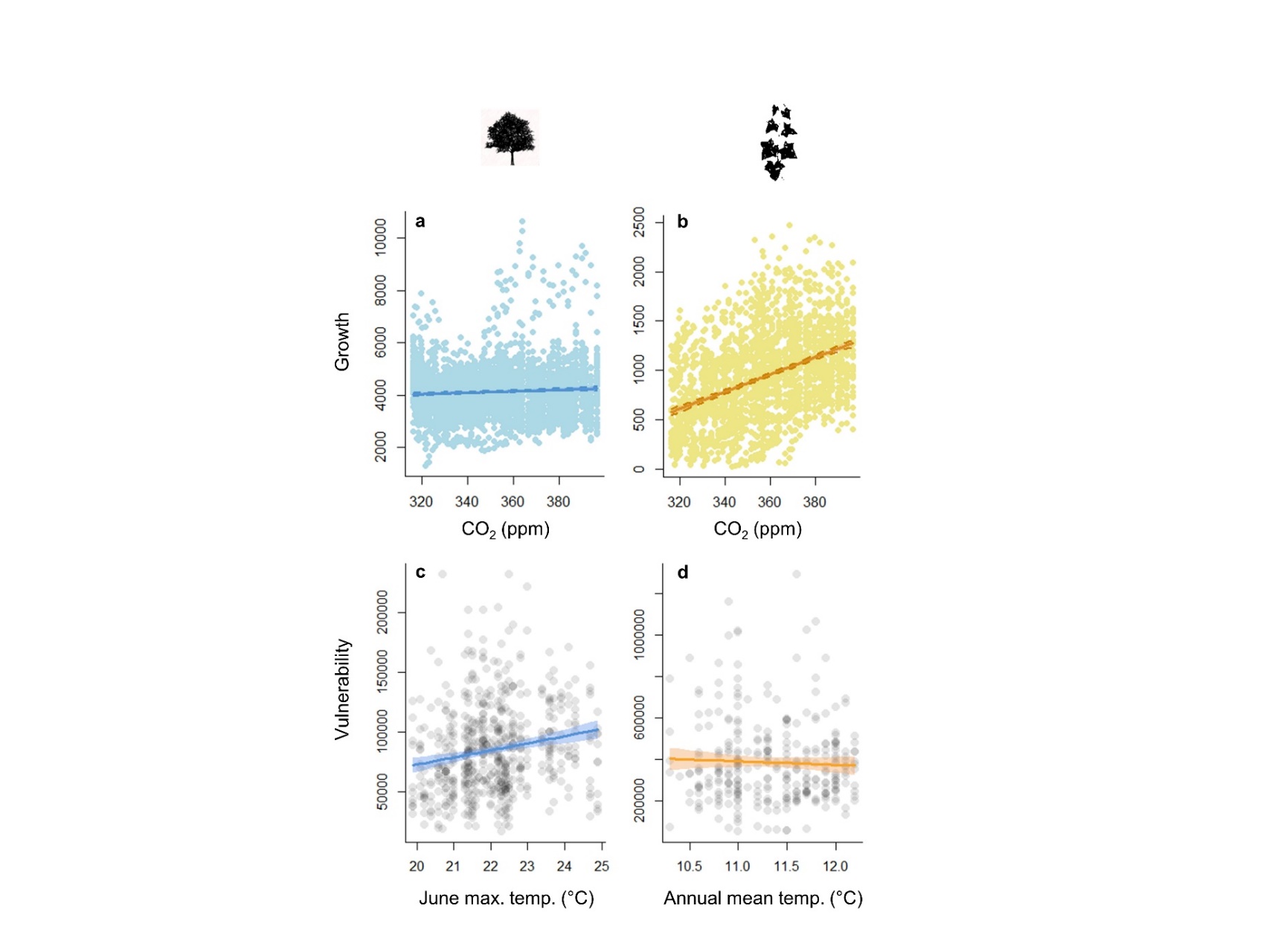


**Fig. S3** Raw correlations between CO_2_ concentration and growth (**a, b**) and between the vulnerability to cavitation and the related temperature variables (**c, d**) for *Quercus cerris* (blue) and *Hedera helix* (orange). GAM and GLMM models are displayed with 95% confidence intervals. Trends are significant (p<0.05) for **a**, **b**, and **c**. Growth is measured as squared root transformed basal area increment.

| *Q. cerris*: sqrt (BAI) ~ T_maxJ_ + CO_2_ + A + P_jun_ + T_maxJ_:CO_2_ + A:CO_2_ + P_jun:_CO_2_, random = ~1\| Tree, correlation = corAR() | | | | |
| --- | --- | --- | --- | --- |
| Variable | Estimate | Std. error | t-value | p-value |
| T_maxJ_ | 1110.74 | 167.84 | 6.62 | <0.001 |
| CO_2_ | 105.34 | 11.42 | 9.23 | <0.001 |
| A | 84.99 | 11.30 | 7.52 | <0.001 |
| P_jun_ | 45.38 | 6.07 | 7.47 | <0.001 |
| T_maxJ_:CO_2_ | -3.18 | 0.47 | -6.74 | <0.001 |
| A:CO_2_ | -0.26 | 0.03 | -8.84 | <0.001 |
| P_jun_:CO_2_ | -0.12 | 0.02 | -7.12 | <0.001 |

**Table S2** Minimum models for both species after variable selection. The final minimal models, after variable selection, are shown above in R code for both species. Estimates, standard errors and statistical significance are displayed for the variables remaining in the final model only. T_maxJ_ = maximum temperature in June; T_a_ = mean annual temperature; CO_2_ = atmospheric carbon concentration; A = tree age; P_jun_ = June precipitation; BAI = basal area increment; Tree = tree identity; corAR() = autocorrelation term in the model.

| *H. helix*: sqrt (BAI) ~ T_a_ + CO_2_ + A + T_a_:CO_2_ + A:CO_2_, random= ~1\|Tree, correlation = corAR() | | | | |
| --- | --- | --- | --- | --- |
| Variable | Estimate | Std. error | t-value | p-value |
| T_a_ | 218.88 | 75.49 | 2.90 | <0.01 |
| CO_2_ | 33.29 | 4.98 | 6.68 | <0.001 |
| A | 60.13 | 11.81 | 5.09 | <0.001 |
| T_a_:CO_2_ | -0.62 | 0.21 | -2.97 | <0.01 |
| A:CO_2_ | -0.17 | 0.03 | -5.01 | <0.001 |

*Sampling biases in dendrochronological studies*

A large controversy has emerged in recent years regarding the detection of growth trends in woody species using tree-ring records (Bowman *et al.* 2012; Brienen *et al.* 2012; Brienen *et al.* 2016). Those works have shown that classical dendrochronological sampling strategies may influence (or completely confound) the detection of meaningful growth trends. Although it is unclear yet the consequences of these biases for previous work and how to completely account for it in future research, it is clear that the central aspect of these biases (commonly referred as ‘big-tree selection bias’ and ‘slow-grower survivorship bias’) is the significant relationship between mean tree-growth and age. The reasoning goes as follows: if fast growing trees tend to die younger than slower growing ones, then by targeting large, dominant individuals, we are unintentionally increasing the chances of having a sample composed by a mix of ‘old slow-growers’ and ‘young fast-growers’. The other two groups are either missing (old fast-growers would have already dominated, died, and thus disappeared from the canopy) or remain unsampled (young slow-growers have not achieve dominance yet). If this unbalanced sampling occurs, it can translate into a spurious positive growth trend that can be mistaken for a response to carbon fertilization or changing climate (Brienen *et al.* 2016).


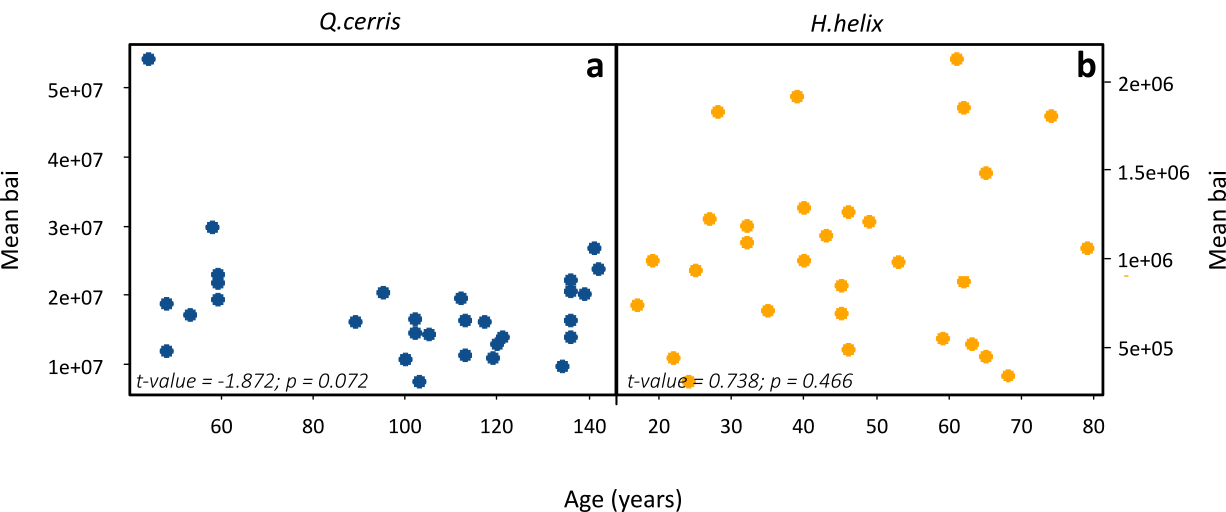
Our sampling strategy focussed on dominant trees, in order to maximize the climatic signal, and could, therefore, be sensible to these biases. However, we found no significant correlation between age and mean growth in our samples (**Fig. S4**), which is, as already mentioned, a necessary requisite for the ‘big-tree selection bias’ and ‘slow-grower survivorship bias’ to occur. As a consequence, we do not expect the abovementioned biases to influence our results.

**Fig. S4** We found no significant relationship between the mean basal area increment (mm^2^*year^-1^) and the tree age (age) for either of our study species.

*Climate change scenarios*

As explained in the main text, we defined climate change scenarios to illustrate the potential consequences of a positive carbon fertilization effect on the predicted distribution of lianas. A key point to consider here, however, is the range of temperatures that are experienced in our study site. Since we found a decreasing positive fertilization effect with higher temperatures, those areas where maximum temperature exceed that of our study place may be misrepresented in our scenarios. Unresponsive curves or even negative carbon effects are possible in those locations. Areas with hotter future temperature than the maximum of our study site (dark grey areas in **Fig. S5a,b**) were located mostly in the southern part of the continent. In these areas, carbon fertilization effects should be further studied, and the carbon fertilization curves can strongly differ from those reported here. However, overlapping these areas with the predicted suitability in **Fig. 5** shows that areas with high uncertainty highly overlap with unsuitable area for *H. helix*, even considering a carbon fertilization effect. We argue, therefore, that this uncertainty does not qualitatively affect our results.


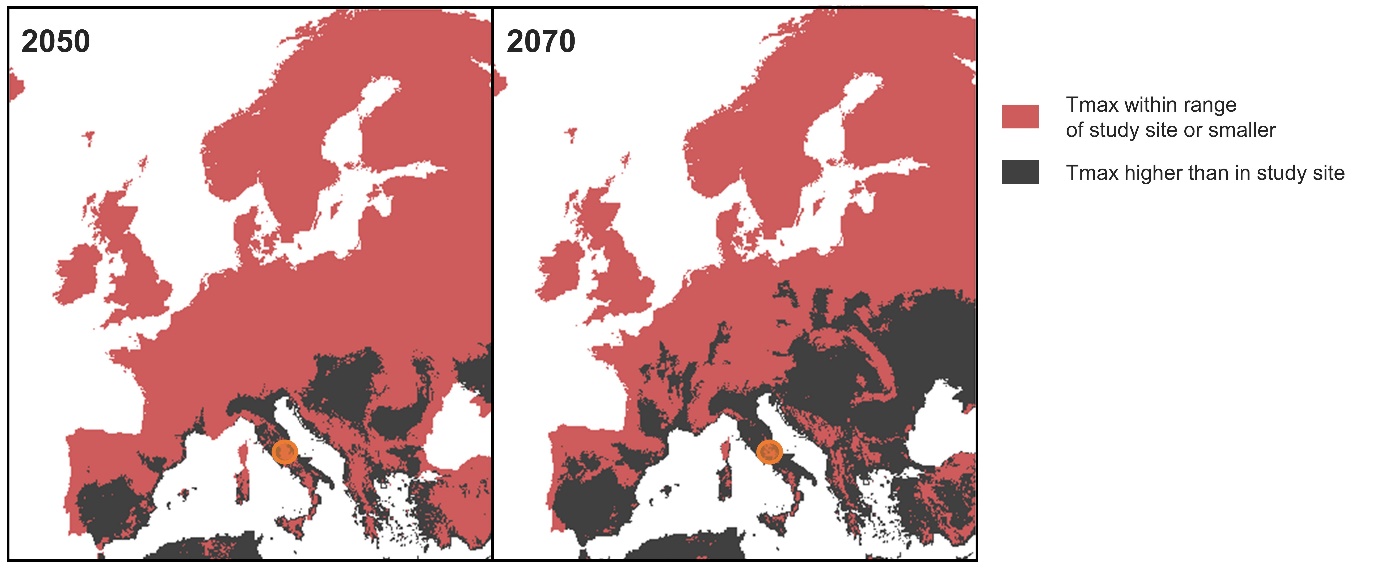
**Fig. S5** Confidence envelope for the fertilization scenarios of Fig. 4. Dark grey areas have a maximum temperatures exceeding the historical range of our study site. For these areas the reported fertilization effect could disappear or even be negative (see Fig. 2). Note that uncertain areas greatly overlap with those for which our models already predict the unsuitable habitat for *Hedera helix* by 2050 and 2070.

**Supplementary references**

Booth, T.H., *et al.* 2014. BIOCLIM: the first species distribution modelling package, its early applications and relevance to most current MAXENT studies. *Diversity and Distributions* 20.1 : 1-9.

Bowman, D.M.J.S. *et al.* 2012. Detecting trends in tree growth: not so simple. – Trends in Plant Science 18: 11–17.

Brienen, R.J.W. *et al.* 2012. Detecting evidence for CO_2_ fertilization from tree ring studies: The potential role of sampling biases. – Global Biogeochemical Cycles, 26, GB1025, doi:10.1029/2011GB004143.

Brienen, R.J.W. *et al.* 2016. Tree demography dominates long-term growth trends inferred from tree rings. – *Global Change Biology* 23: 474-484.

Crawley, M.J. 2013. *The R book, second edition*. Chapter 9: Statistical Modelling - John Wiley and Sons, Ltd.

Hijmans, R.J. 2005. Very high resolution inerpolated climate surfaces for global land areas. – *International Journal of Climatology* 25: 1965–1978.

Jucker, T. 2014. Stabilizing effects of diversity on aboveground wood production in forest ecosystems. Linking patterns and processes. – *Ecology Letters* 17: 1560–1569.

Leuzinger, S. *et al.* 2011. Water relations of climbing ivy in a temperate forest. *Planta* 233: 1087–1096.

Liang, J. *et al.* 2016. Positive biodiversity-productivity relationship predominant in global forests. – *Science* 354: 196–208.

Phillips, S.J. and Dudík, M. 2008. Modeling of species distributions with Maxent: new extensions and a comprehensive evaluation. – *Ecography* 31: 161–175.

Pinheiro, J. *et al.* 2016. nlme: linear and nonlinear Mixed Effects Models. R package version 3.1-128.

Pirone, G. *et al.* 2005. La vegetazione della Riserva Naturale Regionale “Abetina di Rosello” (Abruzzo, Italia). – *Fitosolociologia* 42: 121–137.

Schnitzer, S.A. *et al.* (eds.) 2015. *Ecology of Lianas*. - John Wiley and Sons, Ltd.

Schnitzer, S.A. 2005. A Mechanistic explanation for global patterns of liana abundance and distribution. – *The American Naturalist* 166: 262–276.

Schweingruber, F.H. 1966. *Tree rings and environment dendroecology*. - Paul Haupt.

Speer, J.H. 2010. *Fundamentals of tree-ring research*. - University of Arizona Press.
